# Three-Dimensional Printable Ball Joints with Variable Stiffness for Robotic Applications Based on Soft Pneumatic Elastomer Actuators

**DOI:** 10.3390/polym14173542

**Published:** 2022-08-29

**Authors:** Jin Guo, Jin-Huat Low, Jun Liu, Yangfan Li, Zhuangjian Liu, Chen-Hua Yeow

**Affiliations:** 1 School of Life Science, Beijing Institute of Technology, Beijing 100081, China; 2 Department of Biomedical Engineering, National University of Singapore, Singapore 119077, Singapore; 3 Institute of High Performance Computing, A*STAR Research Entities, Singapore 138632, Singapore

**Keywords:** variable stiffness ball joints, selective laser sintering technology, soft pneumatic elastomer actuators, finite element analysis

## Abstract

This paper contributes to a new design of the three-dimensional printable robotic ball joints capable of creating the controllable stiffness linkage between two robot links through pneumatic actuation. The variable stiffness ball joint consists of a soft pneumatic elastomer actuator, a support platform, an inner ball and a socket. The ball joint structure, including the inner ball and the socket, is three-dimensionally printed using polyamide−12 (PA12) by selective laser sintering (SLS) technology as an integral mechanism without the requirement of assembly. The SLS technology can make the ball joint have the advantages of low weight, simple structure, easy to miniaturize and good MRI compatibility. The support platform is designed as a friction-based braking component to increase the stiffness of the ball joint while withstanding the external loads. The soft pneumatic elastomer actuator is responsible for providing the pushing force for the support platform, thereby modulating the frictional force between the inner ball, the socket and the support platform. The most remarkable feature of the proposed variable stiffness design is that the ball joint has ‘zero’ stiffness when no pressurized air is supplied. In the natural state, the inner ball can be freely rotated and twist inside the socket. The proposed ball joint can be quickly stiffened to lock the current position and orientation of the inner ball relative to the socket when the pressurized air is supplied to the soft pneumatic elastomer actuator. The relationship between the stiffness of the ball joint and the input air pressure is investigated in both rotating and twisting directions. The finite element analysis is conducted to optimize the design of the support platform. The stiffness tests are conducted, demonstrating that a significant stiffness enhancement, up to approximately 508.11 N·mm reaction torque in the rotational direction and 571.93 N·mm reaction torque in the twisting direction at the pressure of 400 kPa, can be obtained. Multiple ball joints can be easily assembled to form a variable stiffness structure, in which each ball joint has a relative position and an independent stiffness. Additionally, the degrees of freedom (DOF) of the ball joint can be readily restricted to build the single-DOF or two-DOFs variable stiffness joints for different robotic applications.

## 1. Introduction

Controllable stiffness is a valued function in various robotic applications, including surgical robotics [1,2,3], walking robots [4,5,6], rehabilitation robotics [7,8] and wearable robots [9,10,11]. Although such compliant mechanisms may result in oscillations and make positioning control of the end effector more difficult, the addition of compliance can offer a number of valuable advantages, including safe interaction between the robot and human, the ability to store and release energy, greater shock tolerance and better adaptability to various unpredictable environments [12,13].

Controllable stiffness mechanisms have been widely applied to both conventional rigid-bodied robots and soft-bodied robots fabricated with soft elastomeric materials with low Young’s modulus. The working principles of the existing variable stiffness designs for conventional rigid-bodied robots can be divided into four groups, including equilibrium-controlled stiffness, structure-controlled stiffness, mechanically controlled stiffness and antagonistic-controlled stiffness [14]. Soft robotics is a rapidly growing research field due to its inherent advantages, such as soft interaction with surrounding environments and compliance for manipulating objects with unknown geometry [15,16,17,18]. The ability to modulate the stiffness of soft actuators is a highly desirable characteristic that allows soft robots to have reversible changes between the compliant/flexible state and the rigid state for various different applications [19,20,21,22,23,24,25]. Smart materials (e.g., electrorheological/magnetorheological fluids [26,27], shape memory polymer [28,29], shape memory alloy [30,31] and low-melting-point alloy [32]), layer jamming [33,34,35] and particle jamming [36,37,38]) have been commonly applied to realize the stiffness modulation of soft robots.

Variable stiffness joints are receiving great attention due to rising interest in the research field of hyper-redundant robotic arms in recent years. Redundant robotic arms are very useful in a highly constrained space or multi-obstacle environments [39]. The redundant robotic arms combined with variable stiffness joints can freely move and adapt to different working environments with low stiffness and withstand external loads with high stiffness when needed. Ball joints are well-suited to be used as modular parts for manipulators due to their multiple degrees of freedom (DOF), excellent positional control characteristic and good structural integrity [40]. Thus, the ball joints with controllable stiffness functionalities have been applied to hyper-redundant robots. To realize the variable stiffness in the ball joint-based mechanism, a few methods have been reported in the literature. Yang et al. [12] proposed a ball joint with variable stiffness based on shape memory polymer (SMP). The SMP is a kind of smart material and is capable of generating a large rigidity range between the soft state and the rigid state by controlling the thermal energy. The ball joints can work with low stiffness when the SMP is heated above its glass transition temperature and increase the stiffness when the SMP is turned into its glass state. However, the heat-sensitive function material requires a relatively long heating time for actuating. Boehler et al. [41] proposed a variable stiffness ball joint for an MR-compatible robot based on a tendon-driven mechanism. Redundant cables have to be tensioned to generate antagonistic forces in the cables for providing the ability of variable stiffness. Additionally, the cables were required to be kept in tension to maintain the rigidity of the joint. Wei et al. [42] presented a stiffness tunable ball joint based on particle jamming. Particle jamming is a promising solution for stiffness modulation by controlling the vacuum pressure because of its simple operation and quick response. Its variable stiffness mechanism depends on the ball joint friction and the compressive force generated by the interaction between the particles and the ball joint. The joint can be rotated when there is no vacuum pressure. When the vacuum pressure is applied, the pressure difference between the inner space and the atmospheric pressure pressed the particles to the joint, thereby forming the jamming. The rotation of the joint is restrained by the jammed particles. However, it is challenging work to choose the suitable particle size. Extra experiments are required to determine the optimal particle size according to different designs. Additionally, the unpredictable particle rearrangement under vacuum pressure should be handled in order to guarantee stability and repeatability.

Different from the currently existing research achievements on the ball joints with controllable stiffness, a new design of variable stiffness ball joints (VSBJ) is proposed to realize the ability of stiffness modulation for ball joints based on pneumatic actuation in this research. A support platform is designed to connect the inner ball to its socket upon pressurization. When the pressurized air is supplied, the soft pneumatic elastomer actuator is inflated to push the support platform against the inner ball, thereby restricting the rotation of the inner ball inside its socket. Thus, the stiffness of the ball joint can be modulated by the input of pressurized air. The contributions of this research and the key features of the proposed VSBJ are summarized as follows: (1) Simple structure: The ball joint is manufactured as an integral mechanism without the requirements of redundant fastening components, which allows the miniaturization design owing to the SLS technology; (2) fast response: The stiffness of the VSBJ can be suddenly enhanced once the pressurized air is supplied to the soft pneumatic elastomer actuator; (3) ‘Zero’ stiffness: The VSBJ can be freely rotated with very little force in its natural state when no air pressure is supplied; (4) positive pressure actuation: Compared with the variable stiffness mechanisms actuated by the smart materials, the pneumatically actuated stiffness modulation proposed in this research is not affected by magnetic/electrical fields or temperature fluctuation. Compared to the variable stiffness mechanisms actuated by vacuum pressure (e.g., particle jamming), the VSBJ actuated by the positive pressure has a wide range of actuation pressure; (5) MRI (magnetic resonance imaging) compatibility: The materials for fabricating the components of the VSBJ (polyamide 12 and silicone elastomer materials) and the actuation mode for the variable stiffness mechanism possess the feature of MRI compatibility [43]. Thus, the proposed VSBJ is able to be used to build variable stiffness structures in the MRI environment. The stiffness variations in both rotating and twisting directions are investigated with respect to different input air pressures. Additionally, the design of the support platform is optimized using finite element analysis. We also demonstrate that multiple VSBJs can be easily combined to build the three-dimensional spatial variable stiffness structures or customized to form the two-dimensional planar variable stiffness joints by limiting the redundant DOFs of the VSBJ.

## 2. Materials and Methods

### 2.1. Design and Working Principle of the VSBJ

**Figure 1:**
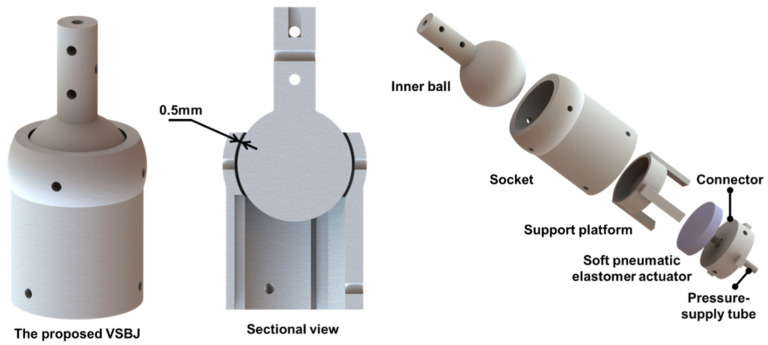
The proposed ball joint with variable stiffness.

The design of the proposed VSBJ is schematically illustrated in Figure 1. The proposed VSBJ includes an inner ball, a socket, a soft pneumatic elastomer actuator, a support platform and a connector. The inner ball and the socket are 3D printed as an integral structure based on PA2200 Nylon material (EOS GmbH, Krailling, Germany) using an EOS P396 3D printer. Polyamide−12 is one of the most well-known and resourceful thermoplastic polymer materials used by SLS technology in the field of additive manufacturing. PA2200 is a kind of thermoplastic semi-crystalline polymer material developed based on PA12 by EOS Company, which possesses a high level of biocompatibility and physical, chemical and mechanical properties [44,45,46,47]. During the SLS process, the inner ball and socket are manufactured in one piece and the powders in the clearance between them are not sintered, just supporting the printed parts. The powders can be cleared after printing and then the inner ball can be freely rotated inside the socket. This fabrication technology effectively simplifies the structure of the VSBJ because redundant fixtures are not required. In conventional ball joint designs, the socket is commonly fabricated into two or more subparts. The subparts and the inner ball are then assembled together to form the ball joint structure by the fastening components. Additionally, we investigated the minimum clearance between the inner ball and its socket, which can ensure the success of manufacturing. It is difficult for the support powders to be cleared with a clearance of smaller than 0.5 mm, which negatively affect the performance of the VSBJ. Thus, the clearance of 0.5 mm is selected in our case.

**Figure 2:**
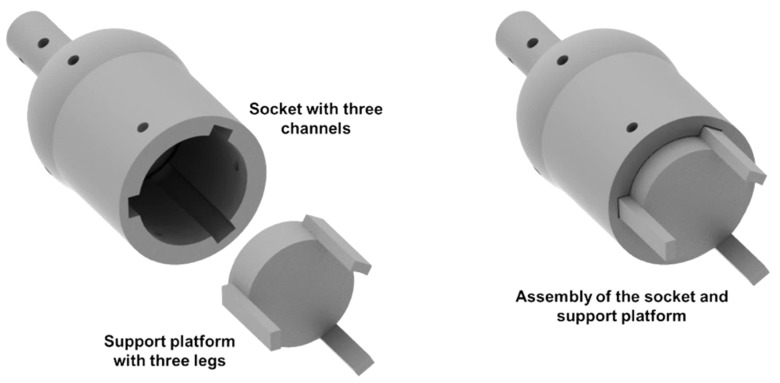
The schematic illustration of the assembly of the socket and support platform.

The socket of the VSBJ is designed with three channels, allowing the support platform to linearly move inside the socket, while limiting the rotation of the support platform (illustrated in Figure 2). The support platform is designed with a groove structure on top and three equally spaced legs positioned in a radial arrangement. The support platform is employed to enhance the stiffness of the ball joint structure by increasing the frictional force between the socket and the inner ball. Three legs of the support platform match the three channels in the socket of the VSBJ. The support platform can be inserted into the socket along the channels from the bottom. The groove structure is employed to contact the inner ball with one stress concentration edge. In the initial state, the inner ball is not pinched by the support platform and the socket. The VSBJ works in its ‘zero’ stiffness state. It can be rotated as the conventional ball joint.

The connector is used to close the socket of the VSBJ. It can be redesigned to connect to other VSBJs or links and is rigidly fixed to the socket. The pneumatic elastomer actuator is positioned between the support platform and the connector. It is made of Ecoflex 0030 (Smooth-On Inc., Macungie, PA, USA, 1:1 parts A:B) that is a very soft platinum cure silicone elastomer with a durometer hardness of Shore 00–30 and high elongation at break (approximately 900%) [48]. The soft pneumatic elastomer actuator is placed in the fully closed space created by the socket, the connector and the support platform. Its expansion can be limited by these three rigid parts. Thus, the soft elastomer actuator can be inflated with much higher air pressure without burst risk compared to the unrestricted soft pneumatic actuators. Although pneumatic actuation is applied to operate the VSBJ, hydraulic actuation is also feasible to inflate the soft elastomer actuator.

**Figure 3:**
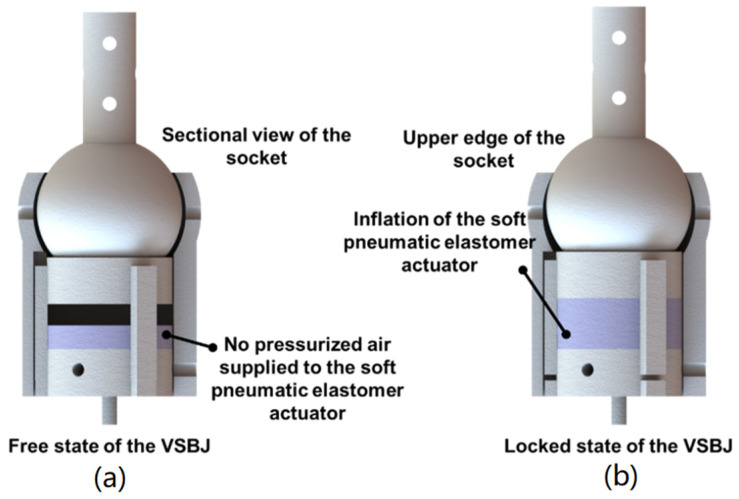
Schematic illustration of the working principle of the VSBJ: (**a**) The free state of the VSBJ; (**b**) the locked state of the VSBJ.

The working principle of the proposed VSBJ is illustrated in Figure 3. In Figure 3a, the VSBJ works in the free state (no pressurized air) and can be freely rotated or twisted. When the pressurized air is supplied, the soft pneumatic elastomer actuator is inflated to compress the support platform against the inner ball (shown in Figure 3b). The stiffness of the VSBJ can be suddenly enhanced due to the fast response of the soft pneumatic elastomer actuator upon pressurization. As illustrated in Figure 3b, the VSBJ works in the locked state, in which the inner ball is pinched by the upper edge of the socket and the support platform, due to the compressing force generated by the inflated soft pneumatic elastomer actuator. The inner ball is restrained from moving by the friction between the inner ball and the socket and the friction between the inner ball and the support platform. The friction is dependent on the input pressurized air. Thus, the higher the air pressure is supplied, the greater is the stiffness that can be obtained.

**Figure 4:**
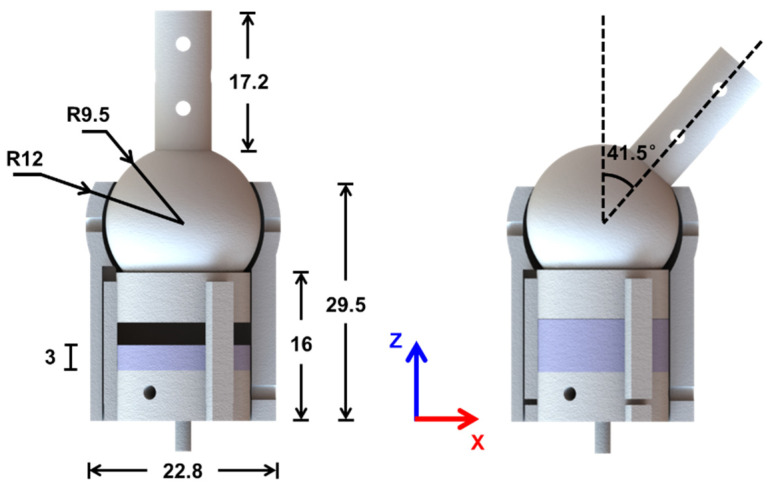
The dimensions in millimeters and rotational range of the VSBJ.

The dimensions of the proposed VSBJ are illustrated in Figure 4. The width, height and diameter of the hollow cylinder of the socket are 24 mm, 29.5 mm and 22.8 mm, respectively. The height of the rod and diameter of the inner ball are 17.2 mm and 9.5 mm, respectively. The heights of the support platform and the soft pneumatic elastomer actuator are 16 mm and 3 mm. The weight of the whole VSBJ is 17.3 g. The inner ball can be rotated around the X and Y axis ranging from −41.5 degrees to 41.5 degrees, respectively, and its twisting motion is unlimited.

### 2.2. Finite Element Analysis of the Support Platform

**Figure 5:**
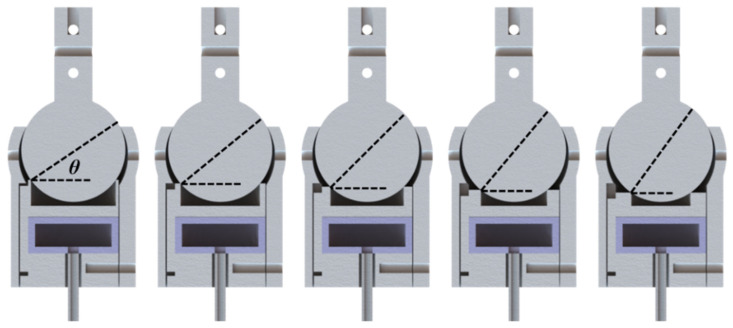
Five support platform designs with increasing intersection angles (θ) assembled with the ball joint structure (Sectional view).

**Figure 6:**
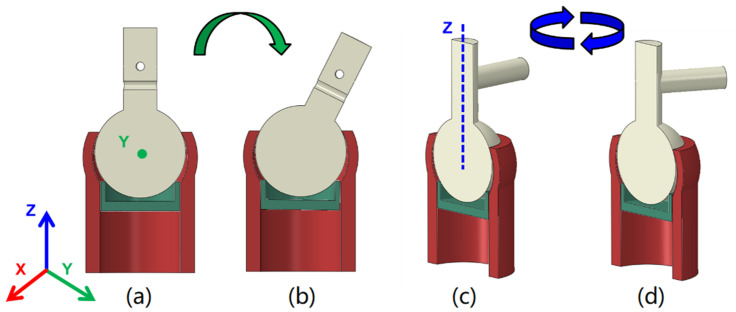
Simulation setups for the VSBJ: (**a**) Initial state for rotating test. (**b**) Final state for rotating test. (**c**) Initial state for twisting test. (**d**) Final state for twisting test.

In addition to the input air pressure, the support platform also plays an important role in VSBJ stiffness when the ball joint structure is determined and manufactured. The design of the support platform was optimized based on finite element analysis by investigating the relationship between the VSBJ stiffness and the intersection angle (θ) (illustrated in Figure 5). Five support platform models with increasing intersection angles, including 35.3 degrees, 40.3 degrees, 45.3 degrees, 50.3 degrees and 55.3 degrees, were built using Solidworks (Dassault Systèmes SolidWorks Corp., Waltham, MA, USA). The 3D models of the VSBJ were imported into ABAQUS/Standard (Dassault Systèmes Simulia Corp., Johnston, RI, USA) to analyze the stiffness enhancement in both rotational and twisting directions using these five support platforms at the air pressure of 100 kPa. In the simulation, the ball joint structure was modeled using hexahedral hybrid elements (Abaqus element type C3D8H) which is an 8-node hybrid element. Since the focus of the simulation was to analyze the behavior of the inner ball and the influence of different platform designs with different intersection angles, the model was simplified by removing the soft pneumatic elastomer actuator and unimportant features, and the actuation pressure was directly applied on the bottom surface of the platform. For this simplified model, ENCASTRE boundary condition was employed on the socket to fix the entire device. The element size is around 0.25 mm, yielding a total number of ~390 K elements for the entire model. Two contact pairs were defined for describing the contact behavior between the inner ball surface and two edges on the socket and the support platform, respectively. During the simulation, the platform was first actuated in the first step, and pushed the inner ball to the stable state where it closely contacted with both the socket and the support platform. This step was followed by actuating the inner ball with either a rotating or twisting force to make the inner ball rotate/twist. For all the loading steps, the dynamic analysis was adopted to consider the effect of sliding friction. The reaction force on the loading point is recorded at different rotating/twisting angles to evaluate the performance of the VSBJ. The initial and final states for rotating and twisting tests were illustrated in Figure 6.

The simulation results were shown in Table 1, demonstrating that the stiffness of the VSBJ in both rotational and twisting directions was dependent on the intersection angle when the same air pressure was supplied. The smaller the intersection angle, the larger stiffness enhancement the VSBJ obtained. Therefore, taking into consideration the material strength and the size of the groove structure, the support platform with the intersection angle of 40.3 degrees was determined in our final design of the VSBJ.

### 2.3. Fabrication of the Soft Pneumatic Elastomer Actuator

**Figure 7:**
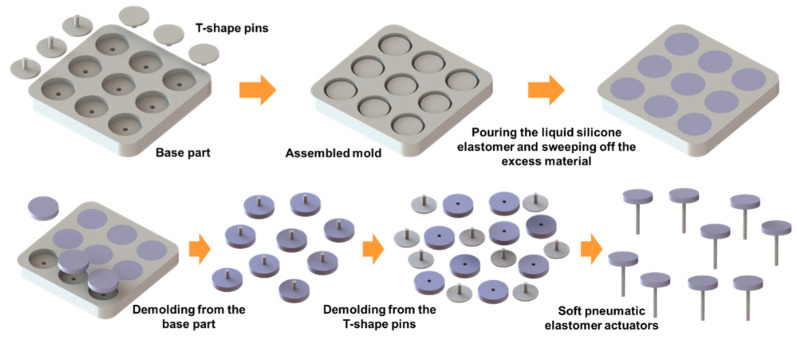
The fabrication process of the soft pneumatic elastomer actuators.

The soft pneumatic elastomer actuator should be capable of withstanding much higher air pressure to achieve better stiffness enhancement. Thus, the body of the soft pneumatic elastomer actuator is required to be fabricated in one piece without any seams, which may be structurally weak and are prone to delamination. Ecoflex 0030 is chosen to fabricate the soft pneumatic elastomer actuator because it is highly extensible with low actuation pressure. The molding method was applied and the fabrication process was illustrated in Figure 7. The mold consisted of two parts: the base part for achieving the actuator bodies and the T-shape pins for obtaining the pressure chambers. The T-shape pins were first inserted into the holes of the base part, ensuring that the bottom of the pins was aligned to the bottom of the base part. Scotch tapes were then applied to the bottom of the base part to avoid the leakage of the liquid silicone elastomer through the clearance between the pins and holes. Next, parts A and B of the liquid silicone elastomer were dispensed into a container according to 1A:1B by weight. The silicone elastomer in the container was mixed well by using a planetary centrifugal mixer. After that, the mixed silicone elastomer was slowly poured into the mold, making sure that all the gaps between the base part and pins were filled up. Vacuum degassing was applied to eliminate the entrapped air in the liquid silicone elastomer. A piece of acrylic sheet with the straight edge was used to sweep off the excess liquid silicone elastomer on the mold while the silicone elastomer inside the gaps remained intact. Finally, the mold was placed in the oven at the temperature of 70 degrees Celsius until the silicone elastomer was cured.

During the demolding process, the pins enclosed with the soft actuator bodies were detached from the base part. After that, the pin was removed from the actuator body by enlarging the opening owing to the feature of the high elongation at break of Ecoflex 0030. Finally, a pressure-supply silicone tube was inserted into the opening and glued with the actuator body by the Sil Poxy adhesive (Smooth-On, Macungie, PA, USA).

## 3. Results

The stiffness of the VSBJ indicates the ability to maintain the position and orientation of the inner ball against the external force. The variable stiffness feature of the VSBJ is a result of the change of the frictional force that is dependent on the inflation of the soft pneumatic elastomer actuator. To investigate the stiffness variation of the VSBJ in both rotating and twisting directions at different input air pressures, including 100 kPa, 200 kPa, 300 kPa and 400 kPa, for the soft pneumatic elastomer actuator, a series of stiffness measurement experiments were conducted. A compressor was employed to supply the air pressure and a precision regulator (IR2020–02B-A, SMC Corporation, Tokyo, Japan) was used to modulate the supplied air pressure to the soft pneumatic elastomer actuator.

### 3.1. Rotating Stiffness Evaluation

**Figure 8:**
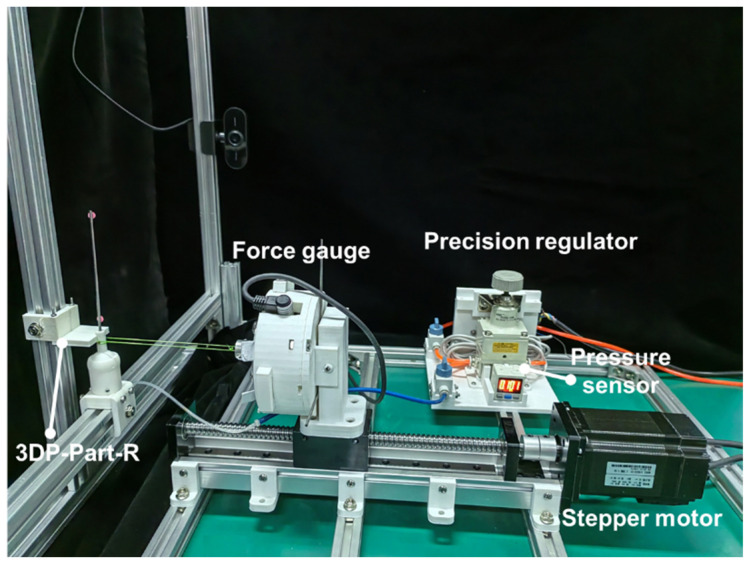
The experimental setup for testing the rotational stiffness of the VSBJ.

The experimental setup for evaluating the rotating stiffness of the VSBJ was shown in Figure 8. The VSBJ was fixed to the aluminum beam structure and a cylindrical rod with two colored markers was inserted into the hole on top of the inner ball. A force gauge (SI-65–5, ATI Industrial Automation Inc., Apex, NC, USA) was fixed on a motorized linear module to measure the pulling force. A short bolt was inserted through the rod of the inner ball and a pulling cord was introduced to connect the short bolt to the force gauge. A high-definition camera was employed to capture the rotational movements of the inner ball and an image processing algorithm was implemented to detect and track the colored markers for obtaining the rotational angles. The pulling cord was pretensioned with 0.2 N and the force gauge was zeroed before each test. In the free state of the VSBJ, the inner ball can be freely rotated inside the socket. It contacted the bottom edge of the socket at its original position when no air pressure was supplied due to its gravity and the existence of the ball joint clearance. Thus, an initial pressure of 11 kPa was provided to inflate the soft pneumatic elastomer actuator, making the upper surface of the inner ball contact the upper edge of the socket. In this situation, the VSBJ had the same rotational center in both the free state and locked state. Therefore, the stiffness of the VSBJ at the pressure of 11 kPa was obtained as the reference value in the free state. The inner ball was positioned at 0 degrees with the assistance of a 3D printed part (3DP-Part-R shown in Figure 8) before each measurement.

**Figure 9:**
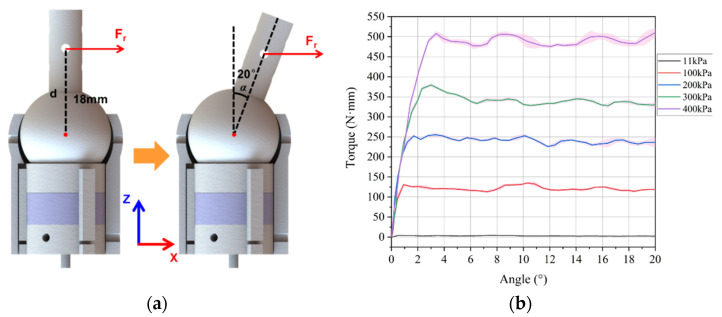
Experimental results of the rotational stiffness of the VSBJ at different input air pressures: (**a**) Schematic illustration of the point of application of the pulling force; (**b**) torque-angle curves at different air pressures.

During the experiments, the soft pneumatic elastomer actuator was inflated with different constant pressures (6 kPa, 100 kPa, 200 kPa, 300 kPa and 400 kPa, respectively). For each input air pressure, the inner ball of the VSBJ was rotated ranging from 0 to 20 degrees by the force gauge through the linear motorized module at 1.25 mm/s constant speed. Fr was the applied pulling force along the X-axis perpendicular to the rod of the inner ball in the initial state, as illustrated in Figure 9a. The distance from the point of application of the pulling force to the center of the ball was 18 mm. The test for each input pressure was repeated five times. The pulling force with respect to the rotational angles was recorded and the obtained data were converted into torque by Equation (1). The mean value (solid line) and standard deviation (shaded area) of the external torque that the VSBJ can withstand in the rotational direction at different input air pressures were illustrated in Figure 9b.

(1) $$T_{r}=F_{r}\cdot d\cdot \cos\alpha$$

As illustrated in Figure 9b, sudden increments in force were observed at the beginning in all the measurement experiments due to the fact that the stiffness of the VSBJ was enhanced by the static friction. After that, the pulling force evidently decreased to a lower value than the peak force because the stiffness of the VSBJ was maintained by the kinetic friction instead of the static friction. Fluctuations in force corresponding to the stick-slip phenomenon were observed during the following rotation. In our study, we defined the maximum torque provided by static friction as the highest stiffness enhancement that the VSBJ can provide. We considered that the VSBJ failed to resist the external load when turning into fluctuations. Experimental results demonstrated that the proposed VSBJ was capable of changing stiffness in the rotational directions within a large range by controlling the supplied air pressure. In the free state of the VSBJ (at the pressure of 11 kPa), the ball joint was able to resist approximately 0.2 N external force around the center of the ball with the moment arm length of 18 mm. Thus, the VSBJ can resist the maximum external torque of 3.6 N·mm in its free state. With the increase in the supplied air pressure, a higher stiffness enhancement can be obtained. At the input pressure of 400 kPa, the VSBJ was capable of resisting the external force of approximately 28.25 N that can lead to the torque of 508.11 N·mm.

### 3.2. Twisting Stiffness Evaluation

**Figure 10:**
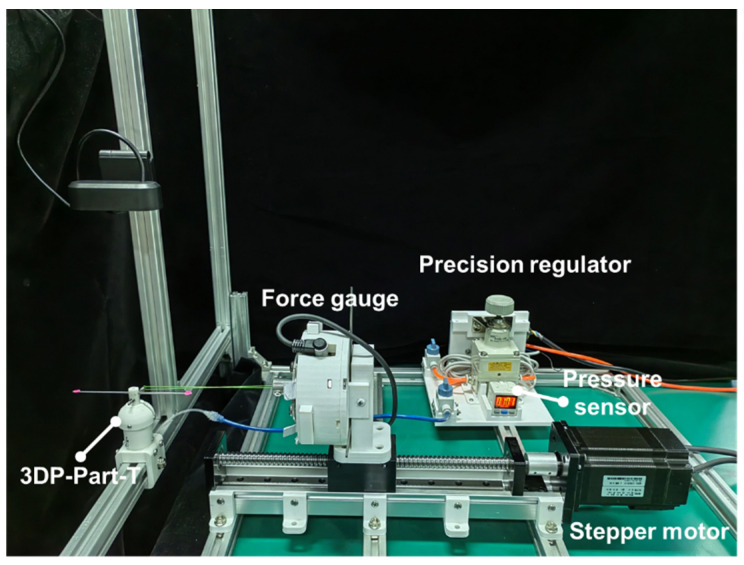
The experimental setup for measuring the twisting stiffness of the VSBJ.

The experimental setup for evaluating the twisting stiffness of the VSBJ was illustrated in Figure 10. The methods of measuring the pulling force and twisting angles were the same as that used in the measurement experiments for rotating stiffness. A 3D printed part (3DP-Part-T shown in Figure 10) was used to allow the inner ball of the VSBJ to twist around the Z axis while limiting its rotations around the X and Y axis. The relationship between the stiffness of the VSBJ in the twisting direction and the supplied air pressure was investigated. Similar to the experiments on rotational stiffness measurement, an initial pressure of 11 kPa was supplied to the soft pneumatic elastomer actuator and the twisting stiffness of the VSBJ at the pressure of 11 kPa was determined as the reference value in the free state.

**Figure 11:**
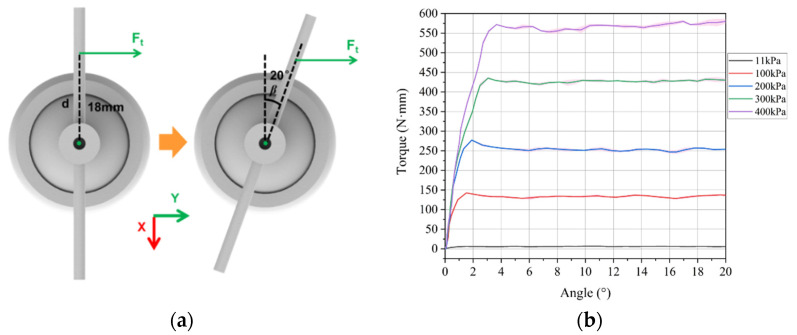
Experimental results of the twisting stiffness of the VSBJ at different input air pressures: (**a**) Schematic illustration of the point of application of the pulling force; (**b**) torque-angle curves at different air pressures.

During the experiments, the VSBJ was twisted ranging from 0 to 20 degrees by the force gauge through the linear motorized module at 1.25 mm/s constant speed. Ft is the applied pulling force along the Y-axis perpendicular to the short bolt inserted through the rod of the inner ball, as illustrated in Figure 11a. The distance from the point of application of the pulling force to the twisting center was 18 mm. The test for each input pressure was repeated five times. The pulling force with respect to the twisting angles was recorded and the obtained data were converted into torque by Equation (2). Figure 11b illustrated the mean value (solid line) and standard deviation (shaded area) of the external torque that the VSBJ can withstand in the twisting direction at different input air pressures from 100 to 400 kPa with an interval of 100 kPa. From the experimental results, sudden increments and fluctuations in force were also observed. Similarly, the maximum torque in the twisting direction provided by the static friction was defined as the highest stiffness enhancement that the VSBJ can provide. The situations that fluctuations happened were considered as the failure for the VSBJ to resist the external load. In the free state, the VSBJ was with the ability to resist approximately 0.32 N external force around the Z axis with the moment arm length of 18 mm. As expected, supplying a higher air pressure led to an increase in the twisting stiffness of the VSBJ. At the input pressure of 400 kPa, the VSBJ was capable of resisting the external force of approximately 31.84 N that can lead to the torque of 571.93 N·mm.

(2) $$T_{t}=F_{t}\cdot d\cdot \cos\beta$$

### 3.3. Customizations of the VSBJ

**Figure 12:**
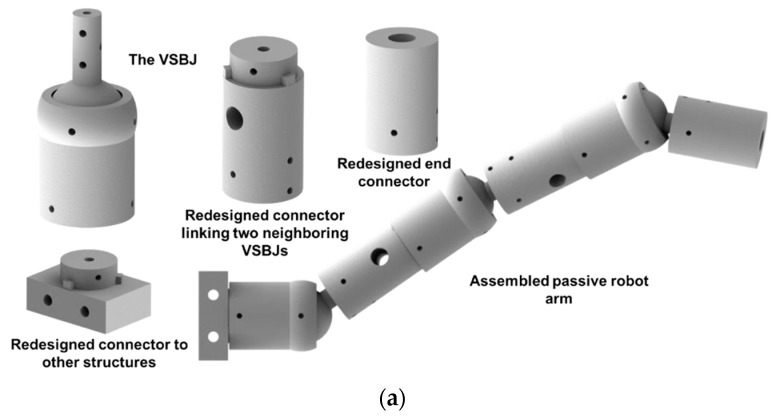

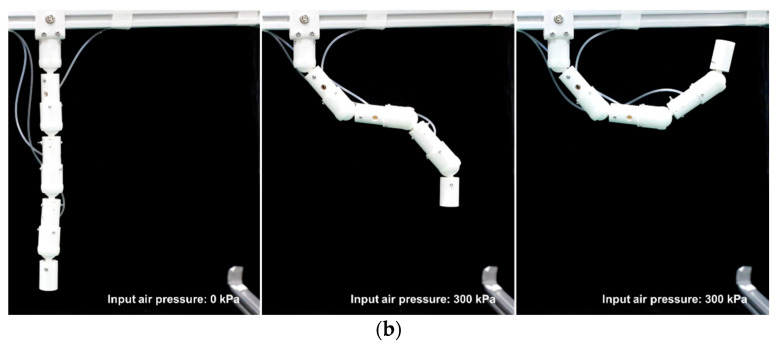
The modularization of the VSBJ: (**a**) Schematic illustration of the redesigned connectors; (**b**) assembled passive robot arm with variable stiffness including four VSBJs and three links.

The proposed VSBJ can be modularized by redesigning the connectors (illustrated in Figure 12a). As illustrated in Figure 12b, four VSBJs and three links were employed to build a passive robot arm. The arm can be lengthened or shortened by modulating the number of joints and links according to different applications. It was capable of being manually moved to the target position in the free state and being quickly stiffened by triggering the variable stiffness mechanisms. Additionally, the stiffness of each VSBJ of the arm allowed to be independently controlled. The manipulation of the passive robot arm was shown in the Supplementary Video S1.

**Figure 13:**
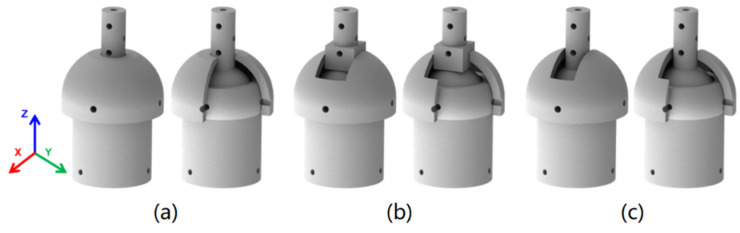
The customized variable stiffness joints based on the VSBJ: (**a**) The variable stiffness twisting joint; (**b**) the variable stiffness revolute joint with one degree of freedom; (**c**) the variable stiffness revolute joint with two degrees of freedom. (The right image in each subfigure shows the sectional view of its corresponding variable stiffness joint).

The VSBJ is with three rotational degrees of freedom around the coordinate axis and it can be easily customized to form the variable stiffness revolute joints with one or two degrees of freedom, as illustrated in Figure 13. Figure 13a showed the twisting joint with variable stiffness. A 3D printed part was positioned on top of the inner ball and fixed with the socket of the VSBJ. It was able to allow the inner ball to twist around the Z axis and restrict the rotational movements around the X and Y axis. Figure 13b presented the variable stiffness revolute joint with one degree of freedom. The rotational movements around the X and Z axis were limited by the 3D printed parts. The revolute joint can only be rotated around the Y axis. Figure 13c illustrated the variable stiffness revolute joint with two degrees of freedom. The revolute joint allowed the inner ball to rotate around the Y axis while rotating around the Z axis. Only the rotation around the X axis was limited in this design. All these joints mentioned above possessed the same variable stiffness mechanism with the VSBJ. The operations of the variable stiffness joints mentioned above were shown in the Supplementary Video S2.

## 4. Conclusions and Future Work

Departing from the existing approaches to ball joints with tunable stiffness functionalities, a new design of the ball joint with variable stiffness is proposed in this research. Owing to the selective laser sintering (SLS) technology, the ball joint structure can be 3D printed as an integral mechanism without the need of assembly, thereby avoiding fastening components. Therefore, the proposed VSBJ can be miniaturized. A support platform was designed and combined with the socket to form the variable stiffness mechanism for resisting the rotational movements of the inner ball. The stiffness of the VSBJ was dependent on the friction force between the socket, the inner ball and the support platform. The design of the support platform was optimized by using finite element analysis. A soft pneumatic elastomer actuator was used to enable the variable stiffness mechanism to be actuated by pneumatic actuation. The stiffness of the VSBJ was dependent on the air pressure supplied to the soft pneumatic elastomer actuator. Experimental results have demonstrated a significant variation in stiffness at different pressure values. The higher stiffness of the proposed VSBJ can be obtained by increasing the supplied air pressure. The proposed VSBJ possessed the key features, including simple structure, fast response, ‘zero’ stiffness, positive pressure actuation and MRI compatibility. The stiffness of the VSBJ was independent on the position of the inner ball relative to its socket. Additionally, the proposed VSBJ can be used as a module for building the variable stiffness arm. It can also be customized to form the variable stiffness revolute joints with one or two degrees of freedom.

The limitation of the proposed VSBJ is the abrasion between the inner ball and the socket due to friction. As for such friction-based stiffness variable mechanisms, abrasion is inevitable. The proposed VSBJ can be connected with links to form a positioning arm as described above. A future extension of this research will be adding the actuation mechanism based on pneumatic actuation for this passive robotic arm.

## Figures and Tables

**Table 1:** The simulation results of the reaction force in both rotating and twisting directions with respect to different intersection angles.

θ	35.3°	40.3°	45.3°	50.3°	55.3°
Rotation	7.76 N	7.57 N	7.28 N	7.15 N	7.02 N
Twist	8.21 N	7.59 N	7.16 N	6.75 N	6.46 N

## Data Availability

The CAD files for the fabrication of the SPEAs presented in this study are available on request from the corresponding author.

## Supplementary Materials

The following supporting information can be downloaded at: https://www.mdpi.com/article/10.3390/polym14173542/s1, Video S1: the manipulation of the passive robot arm including four VSBJs and three links; Video S2: the operations of the customized variable stiffness joints based on the VSBJ.
